# Can Emergency Physicians Diagnose Cirrhosis by Ultrasound: A Prospective Single-Arm Educational Intervention

**DOI:** 10.7759/cureus.38012

**Published:** 2023-04-23

**Authors:** Ashton E Kilgore, Erin F Shufflebarger, Maxwell A Thompson, Mohd Zahid, John P Gullett, David C Pigott, Samuel L Burleson

**Affiliations:** 1 Emergency Medicine, University of Alabama at Birmingham, Birmingham, USA; 2 Radiology, University of Alabama at Birmingham, Birmingham, USA

**Keywords:** medical education, resource-limited setting, emergency physician, ultrasound, cirrhosis

## Abstract

Background and purpose

Liver cirrhosis is common, and timely diagnosis of decompensated cirrhosis may impact acute care and resuscitation. Point-of-care ultrasound is a core competency of US emergency medicine training and is increasingly available in many acute care settings, including those where usual diagnostic modalities of cirrhosis may not be available. Only a few works of literature exist that evaluate the ultrasound diagnosis of cirrhosis and decompensated cirrhosis by emergency physicians (EPs). We aim to evaluate whether EPs can diagnose cirrhosis by ultrasound after a brief educational intervention and determine the accuracy of EP-interpreted ultrasound compared to the radiology-interpreted ultrasound as a gold standard.

Methods

This single-center prospective single-arm educational intervention evaluated the accuracy of EPs diagnosing cirrhosis and decompensated cirrhosis on ultrasound before and after a short educational intervention. Responses were paired across the three assessments, and paired sample t-tests were performed. Sensitivity, specificity, and likelihood ratios were calculated using attending radiology-interpreted ultrasounds as the gold standard.

Results

EPs scored a mean of 16% higher on a delayed knowledge assessment one month after the educational intervention than on the pre-intervention assessment. EP-interpreted ultrasound revealed a sensitivity of 0.90, specificity of 0.71, positive likelihood ratio of 3.08, and negative likelihood ratio of 0.14 compared to radiology-interpreted ultrasound. The sensitivity of our cohort was 0.98 for decompensated cirrhosis.

Conclusions

After a brief educational intervention, EPs can significantly increase their sensitivity and specificity in diagnosing cirrhosis using ultrasound. EPs were particularly sensitive in their diagnosis of decompensated cirrhosis.

## Introduction

A liver disease accounts for approximately two million deaths per year, and cirrhosis is the 11th most common cause of death worldwide [1]. Hepatitis B and C are the predominant causes of cirrhosis worldwide, particularly in low-income countries, followed by alcohol-related liver disease and non-alcoholic steatohepatitis [2].

Decompensated cirrhosis is associated with a tenfold increase in mortality compared to the general population [3]. Patients with decompensated cirrhosis often require extensive resuscitation and are at high risk for complications including variceal bleeding with associated airway compromise and hemorrhagic shock, spontaneous bacterial peritonitis, and hepatic encephalopathy. Therefore, accurate recognition of liver cirrhosis and associated pathophysiologic changes is important in acute care settings such as the ED [3].

A biopsy is the traditional diagnostic gold standard for cirrhosis; however, non-invasive transient elastography is now widely considered the initial test of choice, especially when screening for early fibrosis [4]. Neither modality is available in acute care settings. Ultrasound is recommended as the initial imaging modality when cirrhosis or its decompensation is suspected [4,5] and is much more readily available to most acute care settings than other diagnostic modalities recommended by the American College of Radiology Appropriateness Criteria [6].

Accurate diagnosis of cirrhosis using ultrasound has implications in resource-limited settings where advanced imaging modalities, such as computed tomography, may not be available and where the global mortality from cirrhosis is highest [2]. In these settings, the relatively low-cost and high-yield characteristics of ultrasound make it invaluable [7].

Accurate ultrasound diagnosis of cirrhosis could guide appropriate management of decompensated cirrhosis in both high- and low-resource acute care settings, particularly in settings lacking the modalities typically used to diagnose cirrhosis. A recent trial favorably compared hand-held point-of-care ultrasound (POCUS) by hepatologists to radiology ultrasound, transient elastography, and liver biopsy [8], though decompensated cirrhosis was specifically excluded. However, there is little data on the ultrasound diagnosis of cirrhosis by emergency or acute care physicians. The goal of our study is to evaluate whether emergency physicians (EPs) can recognize cirrhotic echotexture and morphology on ultrasound after a short educational intervention.

## Materials and methods

This was a single-center prospective single-arm educational intervention involving emergency medicine (EM) residents and attending physicians at a large urban academic medical center in the United States. The protocol was reviewed and exempted by the Institutional Review Board, and informed consent was obtained. Participants were given a pre-intervention knowledge assessment (pretest) followed by a one-time lecture, either in person or online. This was immediately followed by a post-intervention assessment (posttest). Approximately one month later, a delayed knowledge assessment (delayed posttest) was administered to evaluate knowledge retention.

**Figure 1:**
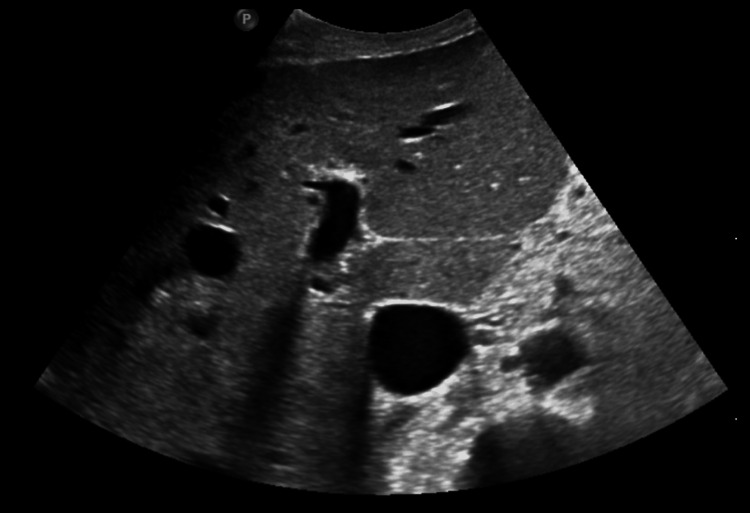
Transverse epigastric view of normal liver

**Figure 2:**
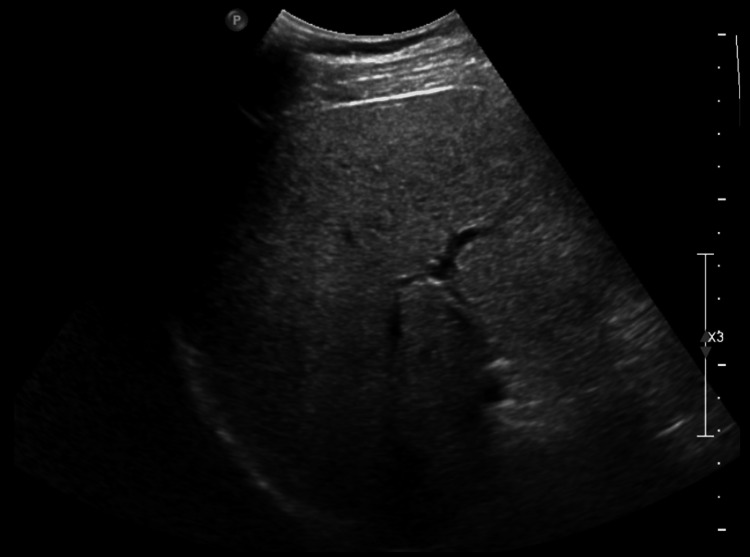
Coronal view of an abnormal, non-cirrhotic (steatotic) liver

**Figure 3:**
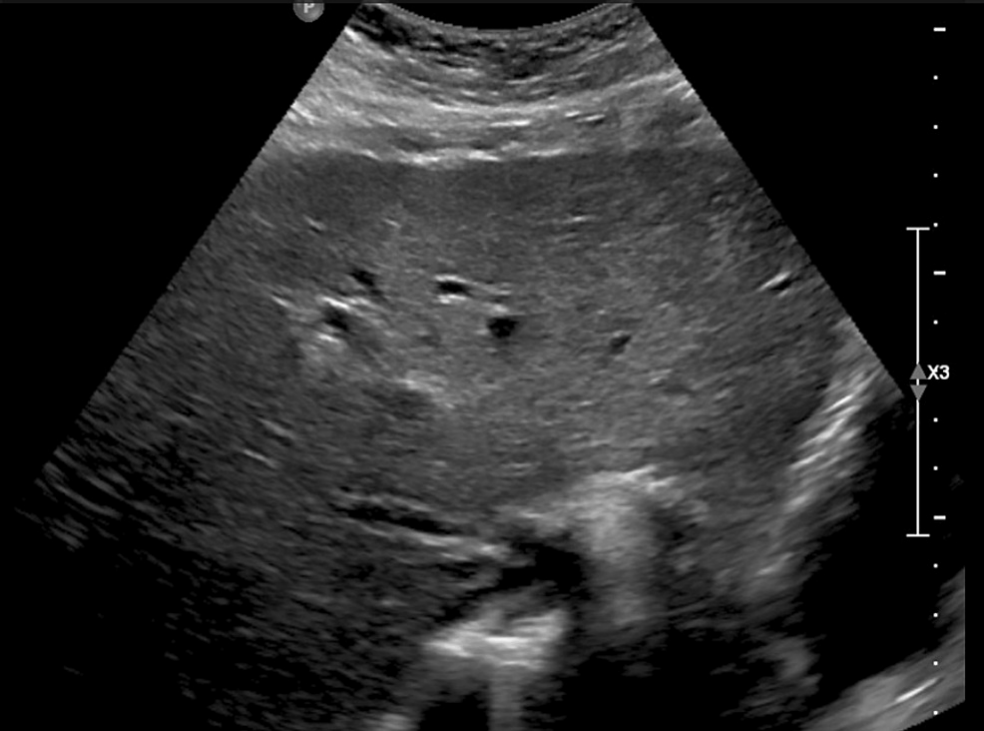
Transverse epigastric view of a cirrhotic liver

**Figure 4:**
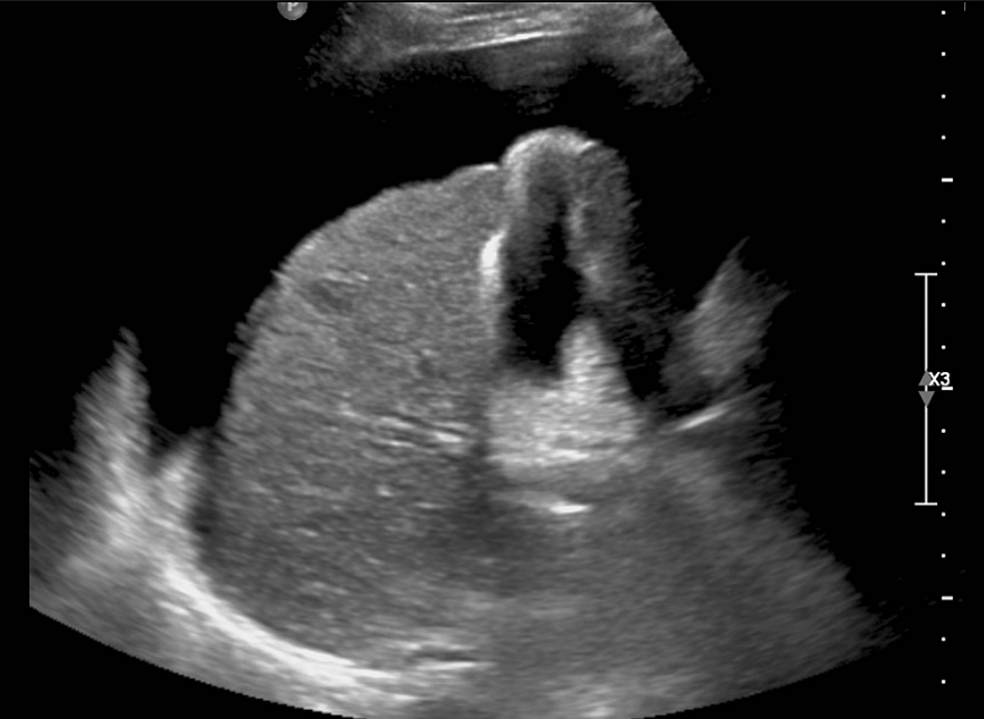
Coronal view of a cirrhotic liver with ascites

**Figure 5:**
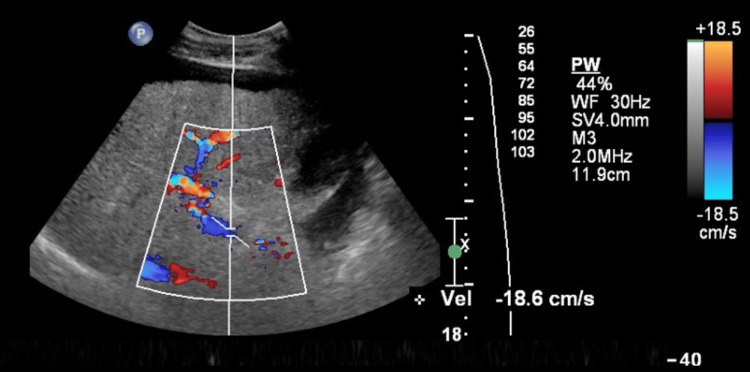
Oblique view of portal vein revealing cirrhosis with reversal of portal venous flow and portal hypertension by color Doppler

The majority of images and clips used in the knowledge assessments were provided by an attending radiologist from the institutional image storage system. These images were from diagnostic ultrasounds previously interpreted by other attending radiologists. Additional images and videos were acquired from established educational image databases and texts [9-12]. The images were sorted according to four categories, as interpreted by the attending radiologist: normal liver, abnormal non-cirrhotic liver, cirrhotic liver, and decompensated cirrhosis (Figures 1-5).

Five images from each of these categories were randomized into each test. The abnormal non-cirrhotic liver images were included to test whether participants could actually detect cirrhosis rather than automatically interpreting any abnormality in the liver as cirrhosis. Three unique 20-question knowledge assessments were created and distributed with Qualtrics (Qualtrics, Provo, UT, USA). Participants were asked to identify whether or not an image demonstrated cirrhosis. Tests may be made available upon request to the corresponding author.

The educational intervention focused on teaching components of cirrhosis and decompensated cirrhosis. The specific components of cirrhosis which were taught included nodular surface, heterogeneous echotexture, shrunken size, and decreased peripheral vascularity, defined subjectively as fewer visible branches of hepatic and portal veins in the liver periphery. Components of decompensated cirrhosis that were taught include ascites and other signs of portal venous hypertension such as splenomegaly, portal venous dilation, and reversal of color Doppler flow in the portal vein. Not all images contained an evaluation of the portal vein or color Doppler flow; however, both normal and abnormal images of each were included.

Participant responses were paired across the three assessments. Data analysis included descriptive statistics and paired sample t-tests (i.e., pretest/posttest, pretest/delayed posttest knowledge assessment comparisons). A p-value of <0.05 was considered significant. All statistical analyses were performed using JMP Pro 16 (JMP Software, Cary NC, USA). Sensitivity, specificity, and likelihood ratios were calculated using attending radiologist-interpreted ultrasounds as the gold standard.

## Results

Twenty-four EPs were enrolled in the study and completed the pretest. Seventeen participants completed the posttest after the educational intervention. After one month, thirteen of the original participants took the delayed posttest. Two participants who took the delayed posttest did not complete the previous posttest, but they had completed the pretest; thus, they were included in the analysis. Three participants who completed the delayed posttest could not be matched to a similar unique identifier from the previous two assessments. Therefore, this data was excluded in the final analysis.

Participants’ scores were significantly higher after the intervention (Table 1). Participants scored 1.83 points, or 9.1%, higher on the posttest when compared to the pretest (t(16)=3.03, p=.0008). Scores on the delayed posttest were 3.37 points, or 16.8%, higher when compared to the pretest (t(12)=8.10, p<.0001).

**Table 1:** Participant knowledge assessment scores *p=.008 compared to pre-score, paired samples **p<.0001 compared to pre-score, paired samples

Knowledge assessment		Assessment score (20 points possible)
Pretest (n=24)		
	Mean ± SD	12.71 ± 2.10
	Median, range	12, 9-16
	Mean % correct	63.55%
Posttest (n=17)		
	Mean ± SD	14.53 ± 1.59*
	Median, range	14, 13-18
	Mean % correct	72.65%
Delayed posttest (n=13)		
	Mean ± SD	16.07 ± 1.44**
	Median, range	16, 13-18
	Mean % correct	80.35%

Immediately after the intervention, the sensitivity, specificity, and positive likelihood ratio of EP diagnosis of cirrhosis by ultrasound all increased, and the negative likelihood ratio decreased (Table 2). Both sensitivity and specificity further increased on the delayed posttest to 0.90 and 0.71, respectively, with a positive likelihood ratio of 3.08 and a negative likelihood ratio of 0.14 (Table 2).

**Table 2:** Sensitivity, specificity, and positive and negative likelihood ratios for EP identification of cirrhosis by ultrasound CI: confidence interval, LR+: positive likelihood ratio, LR-: negative likelihood ratio

	Pretest (95% CI)	Posttest (95% CI)	Delayed posttest (95% CI)
Sensitivity	0.61 (0.55-0.67)	0.78 (0.71-0.84)	0.90 (0.84-0.95)
Specificity	0.66 (0.59-0.72)	0.68 (0.60-0.75)	0.71 (0.62-0.78)
LR+	1.79 (1.46-2.19)	2.40 (1.90-3.03)	3.08 (2.34-4.05)
LR-	0.59 (0.49-0.71)	0.33 (.25-0.45)	0.14 (0.08-0.24)

When stratified by liver pathology, both the sensitivity (Table 3) and specificity (Table 4) of the EPs’ diagnosis of both cirrhosis and decompensated cirrhosis increased after the intervention. The EPs’ sensitivity improved from 0.43 to 0.82 on the delayed posttest, with an improvement in the identification of decompensated cirrhosis from 0.80 to 0.98 (Table 3). The specificity of the diagnosis of normal liver on ultrasound increased from 0.66 to 0.69 on the delayed posttest, with an improvement in the identification of “abnormal but not cirrhosis” diagnosis from 0.66 to 0.72 (Table 4).

**Table 3:** Sensitivity stratified by liver pathology CI: confidence interval

	Pretest (95% CI)	Posttest (95% CI)	Delayed posttest (95% CI)
Cirrhosis	0.43 (0.34-0.52)	0.61 (0.50-0.72)	0.82 (0.70-0.91)
Decompensated cirrhosis	0.80 (0.72-0.87)	0.94 (0.87-0.98)	0.98 (0.92-1.00)

**Table 4:** Specificity stratified by liver pathology CI: confidence interval

	Pretest (95% CI)	Posttest (95% CI)	Delayed posttest (95% CI)
Normal liver	0.66 (0.57-0.74)	0.74 (0.63-0.83)	0.69 (0.58-0.80)
Abnormal (not cirrhosis)	0.66 (0.57-0.74)	0.61 (0.50-0.72)	0.72 (0.61-0.83)

## Discussion

POCUS is a well-established tool for EPs, and competency is a requirement for current EM residency programs approved by the Accreditation Council for Graduate Medical Education (ACGME) in the United States. However, the ultrasonographic diagnosis of cirrhosis is not one of the ACGME core competencies, and there is very little research on EPs’ ability to diagnose cirrhosis using ultrasound. Given the potential for significant management changes based on the presence or absence of cirrhosis, its complications, or its decompensation, we developed this study to determine whether EPs can be taught to identify cirrhosis using ultrasound.

Our results indicate that EPs can be taught to diagnose cirrhosis using ultrasound, and they are more sensitive in identifying decompensated cirrhosis (as evidenced by ascites and other signs of portal venous hypertension such as splenomegaly, portal venous dilation, and reversal of color Doppler flow in the portal vein) than non-severe cirrhosis (Table 3). This may be particularly applicable, as patients with decompensated cirrhosis tend to require the most significant management changes in the ED. An ultrasound-equipped EP or acute care clinician able to accurately diagnose cirrhosis and its decompensation in an undifferentiated critically ill patient or in a low-resource setting will, therefore, be able to tailor resuscitation and narrow the differential diagnosis for that patient earlier in their ED and hospital course. Specific management changes could include the addition of antibiotics and octreotide for upper gastrointestinal bleeding more likely to be variceal in etiology in a newly diagnosed decompensated cirrhotic, or the earlier evaluation and treatment of spontaneous bacterial peritonitis or hepatic encephalopathy in an otherwise undifferentiated patient, all of which decrease morbidity and mortality [3,4,13,14].

Our delayed posttest data (Table 2) compare well with recently published hepatology-performed POCUS data for compensated cirrhosis [8]. That data specifically excludes decompensated cirrhosis, for which our cohort of EPs had a sensitivity of 0.98 (Table 3), preventing true head-to-head comparison.

As a pilot educational intervention study, there are inherent limitations to this research. Most notably, this study assessed image interpretation but did not assess image acquisition. Therefore, this was not a true test of diagnosis of cirrhosis by POCUS. Instead, it evaluated EP interpretation of radiology ultrasound. A more comprehensive study would involve participants both acquiring and interpreting data. However, ultrasound image acquisition is a required skill in current EM residency training in the United States, and EPs view the liver in frequently used required core competencies, such as the Focused Assessment with Sonography in Trauma exam, and evaluate the gall bladder for cholelithiasis or cholecystitis. It follows that EPs with basic ultrasound competency are able to obtain images of liver parenchyma and surface and could readily be trained to assess for portal venous dilation and reversal of color Doppler flow. EPs have demonstrated their ability to apply color Doppler flow in other POCUS applications [15,16]; however, there is currently no data on the use of color Doppler flow in the evaluation of cirrhosis by EPs. The evaluation of the utility of portal venous dilation, splenomegaly, and reversal of color Doppler flow in addition to ascites are potential avenues for further research. Our study did not differentiate between which criterion a participant used to determine whether a liver was cirrhotic by ultrasound.

Our study was limited by sample size. This is a single-center study with a small data set and a single, short educational intervention. Further research on a larger scale in both high- and low-resource settings should be performed to assess the generalizability of our data to other EPs in a variety of settings. Additionally, we lost participants at each stage of evaluation, increasing the risk of attrition bias. We hypothesize that the option to complete the educational intervention and tests asynchronously online may have contributed to attrition over time.

Of interest, the mean score on each assessment increased from the pretest to the posttest to the delayed posttest (Table 1). It is somewhat unclear why, without any further intervention or education, the scores increased from posttest to delayed posttest. It is possible that the EPs who participated were more aware of cirrhosis following the intervention and, as a result, were able to hone their skills over the next month until the delayed posttest was administered. A more likely explanation is that the smaller self-selecting group who completed the study included those with a higher interest and aptitude for ultrasound, resulting in higher overall scores at the end of the study.

We did not differentiate between levels of EM training in this study, which would add an interesting layer for stratification of competency. However, considering our limited sample size, this would have been unlikely to provide meaningful results.

## Conclusions

After a brief educational intervention, EPs significantly increased their sensitivity and specificity in diagnosing cirrhosis using ultrasound. EPs were particularly sensitive in their diagnosis of decompensated cirrhosis. Further research efforts are needed to determine reproducibility on a larger scale and in other settings and EPs' ability to both acquire and interpret images and to determine whether these diagnostic benefits translate to clinically meaningful changes in morbidity and mortality.
